# A Challenging Diagnosis of Papillary Thyroid Carcinoma Arising From Malignant Struma Ovarii

**DOI:** 10.7759/cureus.82465

**Published:** 2025-04-17

**Authors:** Basma Ataallah, Mohammed Al Tameemi, Mustafa Abdulrahman, Zainab Noori

**Affiliations:** 1 Endocrinology, Diabetes and Metabolism, Houston Methodist Hospital, Houston, USA; 2 Endocrinology, Diabetes and Metabolism, Kelsey-Seybold Clinic, Houston, USA; 3 Internal Medicine, Memorial Hermann Greater Heights Hospital, Houston, USA; 4 Internal Medicine, Private Practice, Houston, USA

**Keywords:** germ-cell tumor, malignant struma ovarii, ovarian teratoma, papillary carcinoma of thyroid, thyroid cancer

## Abstract

Ovarian teratomas are a common type of ovarian neoplasm. These tumors have various histologic subtypes, including struma ovarii (SO), which is considered a rare tumor. SO is characterized by the presence of thyroid tissue within its components. Since it contains thyroid cells, it can produce thyroid hormones, leading to thyrotoxicosis, or it can transform into malignant tissue, resulting in thyroid carcinoma, with papillary thyroid cancer being the most common histologic subtype. We present the case of a 46-year-old woman who experienced abdominal pain and was found to have an ovarian mass with malignant features. Excision of the ovaries revealed SO with well to moderately differentiated papillary thyroid cancer. The patient's thyroid function tests were normal, and no distant metastases were found. She was started on a small dose of supplemental thyroid hormone to suppress thyroid-stimulating hormone (TSH) to a low normal level. The highest incidence of malignant struma ovarii (MSO) occurs in the fifth decade of life. The symptoms are usually vague and nonspecific, and the diagnosis is typically made after excision of the adnexal mass. The management of SO is primarily surgical, which is curative in most cases, but should be preceded by tumor staging and postoperative thyroid hormone suppression, along with surveillance using imaging and thyroid biochemical markers.

## Introduction

Struma ovarii (SO) is a rare ovarian dermoid tumor that tends to be a unilateral disease affecting one ovary [1,2]. SO can be either benign or malignant. The majority of patients with SO are asymptomatic. However, some patients can develop symptoms that can be similar in both benign and malignant tumors [3]. The most common presentation based on the literature review is lower abdominal pain, a palpable mass, and ascites, among other nonspecific symptoms [3]. Fewer than 10% of the patients present with hyperthyroidism [4,5]. The diagnosis of malignant struma ovarii (MSO) is usually not straightforward due to poorly defined imaging features, the absence of a specific tumor marker, and the fact that the CA-125 tumor marker is of limited use in such tumors [6]. However, postoperative pathological findings can confirm the diagnosis [6]. The mainstay of management for SO, as with most ovarian tumors, is surgical resection. There are several controversies regarding the surgical approach and postoperative treatment options for patients with MSO. Nevertheless, a multimodal treatment approach for MSO may improve survival rates and reduce the risk of recurrence [7,8]. Due to the rarity of these tumors, we present our case to highlight key points in the clinical and pathological characteristics of these tumors, as well as an outlook on the main management modalities.

## Case presentation

A 46-year-old female patient presented initially to the gynecology clinic for lower abdominal pain accompanied by urine incontinence. A physical exam showed a palpable lower abdominal mass. She underwent a computed tomography (CT) scan of the abdomen and pelvis, which showed a 12.7 cm right ovarian complex cystic lesion with irregular enhancement associated with right hydronephrosis and hydroureter and about a 6 cm complex ovarian cyst on the left ovary. Subsequently, she had a pelvic washing, which was negative for malignant cells.

**Figure 1:**
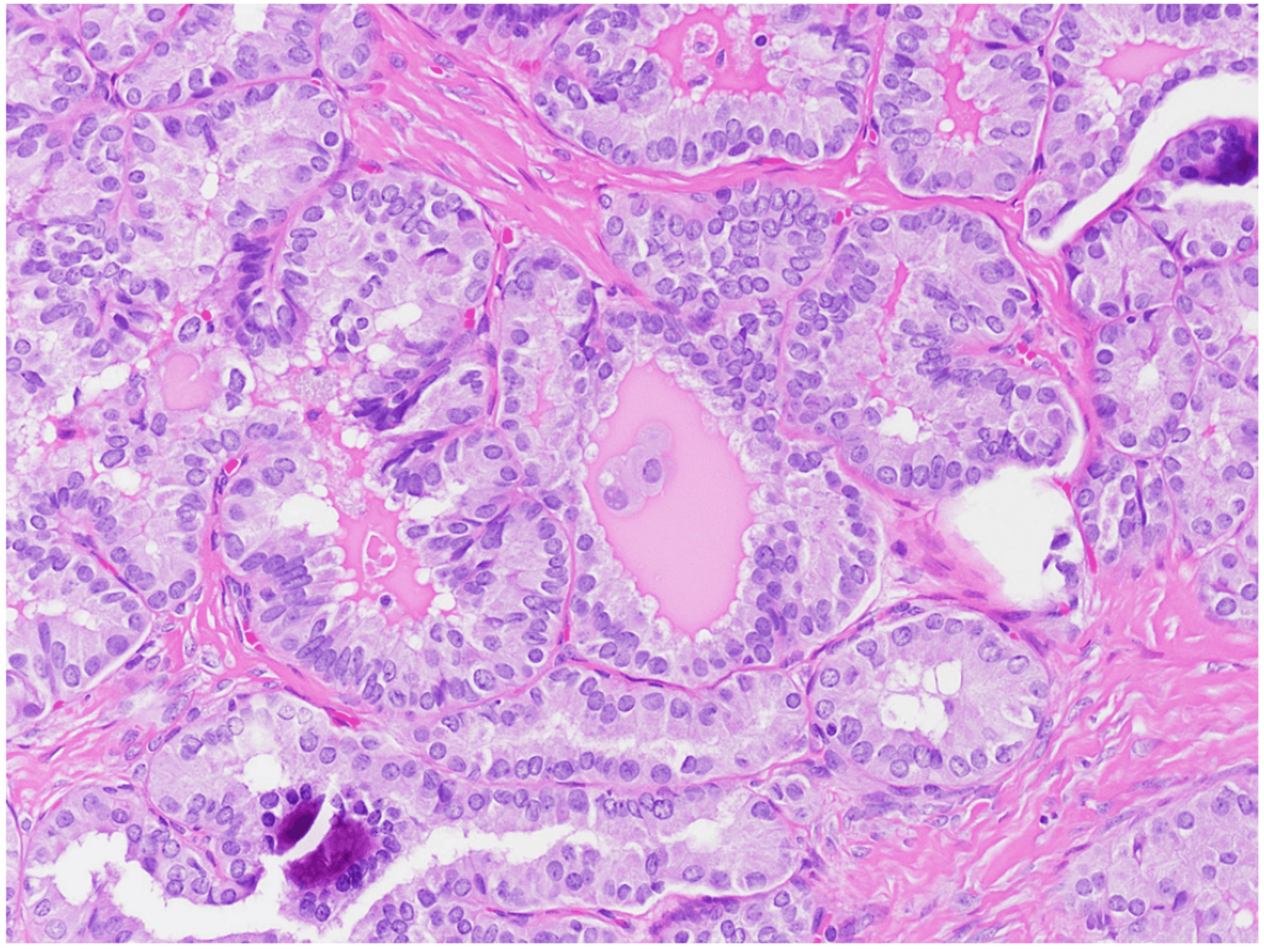
Hematoxylin and eosin section at 200x total magnification showing tumor nuclei enlarged, elongated, and overlapping, with fine powdery chromatin and indistinct nucleoli. Nuclear grooves are noted frequently.

**Figure 2:**
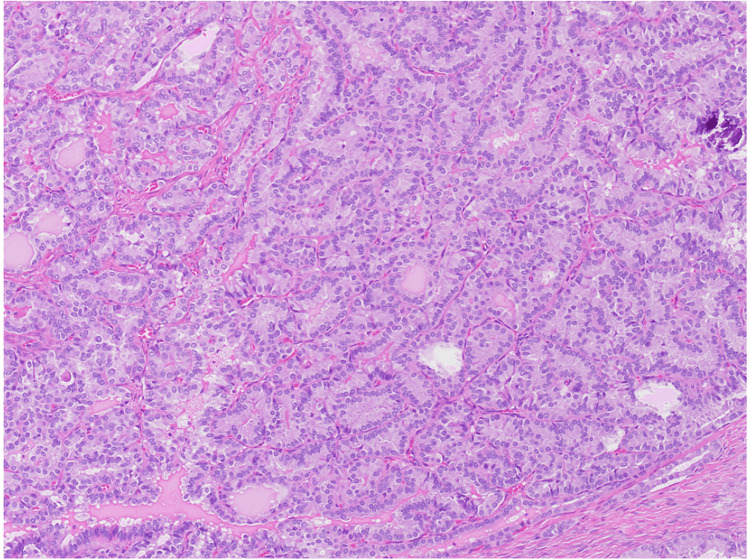
Hematoxylin and eosin section at 100x total magnification showing area of papillary thyroid carcinoma arising in struma ovarii. The papillary fronds are packed together back to back. Psammomatous calcification can be seen on the right.

She then underwent an exploratory laparotomy with total hysterectomy, bilateral salpingo-oophorectomy, a thorough pelvic dissection, and omental biopsy. The pathological finding was compatible with SO, with 18 mm well-to-moderately differentiated papillary thyroid cancer and absence of extraovarian extension (Figures 1-2). Tumor, node, and metastasis (TNM) staging was pT1aNxMx. It was negative for the BRAF mutation. No involvement of the left ovary and fallopian tubes, as well as the uterus, was noted.

The patient denied any family history of thyroid cancer and radiation exposure. Postoperative laboratory values revealed thyroglobulin antigen levels were at 20.1 ng/ml, with undetectable thyroglobulin antibodies. Thyroid-stimulating hormone (TSH) was at 3.5 mIU/L (reference range 0.4-4.5 mIU/L), and free thyroxine (T4) was at 1.1 ng/dl (reference range 0.8-1.8 ng/dL). She also had an ultrasound of the thyroid gland that showed a 6 mm hypoechoic nodular lesion of the right isthmus that did not meet criteria for tissue sampling. 

She was started on levothyroxine to maintain TSH at a low normal level, and she plans to follow up on the small thyroid nodule with ultrasound in three to five years. 

## Discussion

SO is a rare ovarian teratoma that originates from the ovarian germ cell layer. Only 5-15% of ovarian teratomas contain thyroid tissue, and to meet the definition of SO, thyroid tissue should constitute more than 50% of its histology [1]. SO can affect female patients of different age groups, and the incidence is higher in older women. The tumor is usually unilateral, affecting a single ovary, and about 6% of the reported cases are bilateral [2]. SO represents about 1% of all ovarian tumors, and as with other types of ovarian tumors, it is mostly asymptomatic and found incidentally on abdominal or pelvic imaging [3]. Less commonly, patients may present with nonspecific clinical manifestations, such as lower abdominal pain, abdominal mass, ascites, vaginal bleeding, and pleural effusion [3]. Since SO contains thyroid tissue, it can rarely become functional, leading to symptoms of hyperthyroidism, with thyrotoxicosis reported in about 5-8% of the cases [4,5].

The diagnosis of SO is primarily based on postoperative pathological findings, as most patients are asymptomatic or have nonspecific symptoms. Imaging studies like ultrasound, magnetic resonance imaging (MRI), and CT scan aid in diagnosis, while tumor markers lack both specificity and sensitivity [6]. The vast majority of SO cases are benign, with about 5% transforming into malignant tumors [7]. The highest incidence of transformation is reported during the fifth decade of life [7]. Papillary thyroid carcinoma is the most frequently reported type of transformation in these tumors [8]. Less commonly reported thyroid malignancy variants include follicular thyroid cancer, follicular variant of papillary thyroid cancer, and rarely anaplastic or medullary thyroid cancer [8]. The genetic mutations associated with the development of MSO are similar to those found in thyroid gland cancer, with the BRAF (V600E) mutation present in about two-thirds of cases [9]. Other mutations, including KRAS, PTEN, and RET gene mutations, can affect the ovaries similarly to their effect on the thyroid gland [9]. The histopathological diagnostic criteria used postoperatively to confirm the presence of MSO are the same as those used for thyroid gland carcinoma, as reported by Szczepanek-Parulska et al. [10]. However, the clinical behavior of MSO cannot be determined solely based on the histologic features of these tumors. A study by Shaco-Levy et al., who analyzed 86 cases of SO, found no correlation between tumor behavior and microscopic appearance [11]. Distant metastasis of MSO is rare; however, local invasion into adjacent pelvic organs can occur [12]. Thyroid gland cancer can coexist with MSO, and it is recommended to perform imaging studies of the thyroid gland in such patients to rule out simultaneous thyroid gland carcinoma, although this is considered a rare occurrence in these patients [13]. 

The mainstay for the treatment of both benign and malignant SO is surgical resection [14]. Given the rarity of these ovarian tumors, no clear guidelines for optimal management have been proposed thus far [14]. Many experts advocate for conservative pelvic surgery followed by total thyroidectomy and radioactive iodine ablation along with TSH suppression using levothyroxine as the main treatment option for MSO [15]. However, this remains controversial and can be influenced by factors such as tumor extent, the patient's age, and fertility goals [15,16]. Some authors recommend more extensive pelvic surgery for elderly or postmenopausal women and for those without fertility plans [16]. For younger women with fertility concerns, a more conservative pelvic surgery may be recommended, especially given the low tumor recurrence rate; about 7.5% over 25 years in patients with well-differentiated thyroid carcinoma localized to the ovary [17]. Patients with MSO usually have an excellent prognosis, though older age is associated with a higher tumor recurrence rate [18]. The updated American Joint Committee on Cancer TNM staging system for differentiated thyroid cancer suggests a cutoff age of 55 years [19]. 

Postoperative surveillance for MSO is recommended, with measurement of serum thyroglobulin and the use of imaging studies. Prophylactic thyroidectomy followed by radioactive iodine ablation will permit a proper postoperative surveillance plan [16,19].

## Conclusions

Patients with MSO generally have an excellent disease-specific survival rate, regardless of the treatment approach. However, many factors affect the survival rate and can influence the extent of surgical intervention as well as the selection of postoperative treatment options. A multidisciplinary discussion is crucial for tailoring the management, as MSO patients may require additional postoperative treatment and monitoring, and staging is key to assessing whether thyroidectomy and radioactive iodine are needed. Given the rarity of the condition, further studies are needed to establish clear guidelines and more tailored management strategies.
